# Two-stage training algorithm for AI robot soccer

**DOI:** 10.7717/peerj-cs.718

**Published:** 2021-09-17

**Authors:** Taeyoung Kim, Luiz Felipe Vecchietti, Kyujin Choi, Sanem Sariel, Dongsoo Har

**Affiliations:** 1 Cho Chun Shik Graduate School of Green Transportation, Korea Advanced Institute of Science and Technology, Daejeon, South Korea; 2 Department of Computer Engineering, Istanbul Technical University, Istanbul, Turkey

**Keywords:** Multi-agent reinforcement learning, Heterogeneous agents, Centralized training, Deep learning, Robotics

## Abstract

In multi-agent reinforcement learning, the cooperative learning behavior of agents is very important. In the field of heterogeneous multi-agent reinforcement learning, cooperative behavior among different types of agents in a group is pursued. Learning a joint-action set during centralized training is an attractive way to obtain such cooperative behavior; however, this method brings limited learning performance with heterogeneous agents. To improve the learning performance of heterogeneous agents during centralized training, two-stage heterogeneous centralized training which allows the training of multiple roles of heterogeneous agents is proposed. During training, two training processes are conducted in a series. One of the two stages is to attempt training each agent according to its role, aiming at the maximization of individual role rewards. The other is for training the agents as a whole to make them learn cooperative behaviors while attempting to maximize shared collective rewards, *e.g.*, team rewards. Because these two training processes are conducted in a series in every time step, agents can learn how to maximize role rewards and team rewards simultaneously. The proposed method is applied to 5 versus 5 AI robot soccer for validation. The experiments are performed in a robot soccer environment using Webots robot simulation software. Simulation results show that the proposed method can train the robots of the robot soccer team effectively, achieving higher role rewards and higher team rewards as compared to other three approaches that can be used to solve problems of training cooperative multi-agent. Quantitatively, a team trained by the proposed method improves the score concede rate by 5% to 30% when compared to teams trained with the other approaches in matches against evaluation teams.

## Introduction

Recently, deep reinforcement learning (DRL) has been widely applied to deterministic games (Silver et al., 2018), video games (Mnih et al., 2015; Mnih et al., 2016; Silver et al., 2016), sensor networks (Kim et al., 2020), and complex robotic tasks  (Andrychowicz et al., 2017; Hwangbo et al., 2019; Seo et al., 2019; Vecchietti et al., 2020; Vecchietti, Seo & Har, 2020). Despite the breakthrough results achieved in the field of DRL, deep learning in multi-agent environments that require both cooperation and competition is still challenging. Promising results have been for cooperative-competitive multi-agent games such as StarCraft (Vinyals et al., 2019) and Dota (Berner et al., 2019). For multi-agent problems such as multi-robot soccer (Liu et al., 2019), security (He, Dai & Ning, 2015; Klima, Tuyls & Oliehoek, 2016), traffic control (Chu et al., 2019; Zhang et al., 2019), and autonomous driving (Shalev-Shwartz, Shammah & Shashua, 2016; Sallab et al., 2017), non-stationarity, partial observability, multi-agent training schemes, and heterogeneity can be challenging issues (Nguyen, Nguyen & Nahavandi, 2020). To solve these challenges, multi-agent reinforcement learning (MARL) techniques  (Lowe et al., 2017; Sunehag et al., 2017; Foerster et al., 2018; Vinyals et al., 2019; Liu et al., 2019; Samvelyan et al., 2019; Rashid et al., 2020) have been intensively investigated.

When using the MARL, several works have used the centralized training in decentralized execution (CTDE) framework (Lowe et al., 2017; Sunehag et al., 2017; Foerster et al., 2018; Rashid et al., 2020). In the CTDE framework, local observations of agents, global state of the environment, and joint-actions taken by the agents at each time step are available during training to the centralized policy network, while only the local observations of agents are available during execution. In other words, each agent selects its action, that is the output of a policy network, without considering the full information of the environment. To address the non-stationarity problem, multi-agent deep deterministic policy gradient (MADDPG) (Lowe et al., 2017) was proposed using a CTDE framework and the deep deterministic policy gradient (DDPG) actor-critic algorithm for continuous action spaces (Lillicrap et al., 2015). When cooperative behavior is to be achieved, representing that there is a cooperative reward that should be maximized by multiple agents, credit should be assigned accordingly to each agent based on its contribution. To address this problem, counterfactual multi-agent (COMA) (Foerster et al., 2018), value decomposition networks (VDN) (Sunehag et al., 2017), and monotonic value function factorization (QMIX) (Rashid et al., 2020) have been proposed, using the CTDE framework combined with value-based algorithms such as deep Q networks (DQN) (Mnih et al., 2013), deep recurrent Q networks (DRQN) (Hausknecht & Stone, 2015), and dueling Q networks (Wang et al., 2016).

In this paper, a novel training method for MARL of heterogeneous agents, in which each agent should choose its action in a decentralized manner, is proposed. The proposed method addresses how to provide an optimal policy and maximize the cooperative behavior among heterogeneous agents. To this end, during training, two training stages are conducted in a series. The first stage is for making each agent learn to maximize its individual role reward while executing its individual role. The second one is for making the agents as a whole learn cooperative behavior, aiming at the maximization of team reward. The proposed method is designed to be applied to MARL with heterogeneous agents in cooperative or cooperative-competitive scenarios. In this paper, a cooperative-competitive Artificial Intelligence (AI) robot soccer environment is used for experiments. The environment can be described in relation to 5 *versus* 5 robot soccer game described in Hong et al. (2021). In the robot soccer game, two teams of five robots capable of kick and jump behaviors compete against each other, similarly to the StarCraft, so the game can be seen as a micro-management problem. The policy for the proposed method and other methods for comparisons are trained by using self-play (Heinrich, Lanctot & Silver, 2015; Lanctot et al., 2017; Silver et al., 2017). Self-play in a competitive environment is used so that the opponent team is kept at an appropriate level of difficulty at each training stage.

The main contributions of this paper are as follows

1. A framework for novel training method called two-stage heterogeneous centralized training (TSHCT) aiming at centralized training of heterogeneous agents is proposed. In the proposed method, there are two training stages that are conducted in a series. The first stage is responsible for training individual behaviors by maximizing individual role rewards. The second stage is for training cooperative behaviors by maximizing a shared collective reward.
2. Experiments are conducted to compare the performance of the proposed method with other baseline methods, COMA, VDN, and QMIX. The proposed method and the baseline methods are trained with self-play. To compare the results obtained from the experiments, total rewards (during training) and score/concede rates (against different opponent teams) are presented. From the comparisons, we will show better performance of the proposed method during game.
3. The proposed method aims at MARL with heterogeneous agents in cooperative and cooperative-competitive scenarios. For experiments, a cooperative-competitive AI robot soccer environment, where there are 5 robots with 3 different roles in each team (one goalkeeper, two defenders, and two forwards), is used.

The remainder of this paper is organized as follows. ‘Background’ presents the concept of the MARL, system modeling, and other methods which are used as baselines for comparisons in the experiments. ‘Proposed Method’ introduces the proposed method in details. ‘Simulation Results’ presents the simulation environment, ablation studies, and game results of the AI robot soccer. ‘Conclusion’ concludes this paper.

## Background

In this section, the mathematical modeling of the proposed method is presented. Also, other methods for cooperative MARL using the CTDE framework are presented.

### System modeling

The cooperative-competitive multi-agent problem, specifically applied in this paper to AI robot soccer, is modeled as a decentralized partially observable Markov decision process (Dec-POMDP) (Oliehoek & Amato, 2016) that each agent has its own observation of the environment. The Dec-POMDP can be formulated by an 8-tuple *G* =  < *S*, *U*, *P*, *r*, *Z*, *O*, *n*, *γ* >. The set of states and the set of actions are represented by *S* and *U* respectively. Each team contains *n* agents. The observation function *O*(*s*, *a*), where *s* and *a* ∈ {1, …, *n*} are state and *n* agents, determines the observation *z* ∈ *Z* that each agent perceived individually at each time step. At each time step, the *n* agents choose their actions *u*^*a*^ ∈ *U*, which is an action taken by the *a*-th agent, based on their action-observation history. In this modeling, as recurrent neural networks (RNN) (Hochreiter & Schmidhuber, 1997) is used by the MARL algorithm, the policy is conditioned on the joint action-observation history as well as the current agent observation *z*. The state of the environment changes according to a transition probability *P*. Unlike the partially observable stochastic game, all agents in Dec-POMDP share a collective reward and an individual reward drawn from the reward function *r*(*s*, **u**), where **u** is a joint-action which is a set of each agent’s action. The discount factor of the MARL algorithm is represented by *γ*.

In MARL, as multiple agents act simultaneously in the environment based only on their own action-observation history and do not know about the individual policy of each agent, there exists a non-stationarity problem. The behaviors of other agents are changing during training and can influence the reward received by each agent. To address this issue, the system is modeled using a centralized training in decentralized execution (CTDE) framework. In the CTDE framework, the full state of the environment can be accessed in the training procedure to get the state-action value. On the other hand, only the local observation can be accessed by the agent during execution. The joint-action from all agents is also available during the training procedure by the centralized policy to alleviate the non-stationarity issue.

In this paper, we focus on value-based MARL algorithms applied in environments where a sense of cooperation is needed between agents, meaning that they share a collective reward. The proposed algorithm is to be combined with deep recurrent Q-networks (DRQN) (Hausknecht & Stone, 2015) and dueling deep Q-networks (Wang et al., 2016). The DRQN algorithm, as proposed in Hausknecht & Stone (2015), addresses single-agent with partially observable environments. The architecture consists of the DQN (Mnih et al., 2015) combined with RNN. The DRQN approximates the state-action value function *Q*(*s*, *u*), where *s* and *u* are a state and an action of single agent, with RNN to maintain an internal state and aggregate observations over time. It also can be taken to approximate *Q*(*s*_*t*_, *h*_*t*−1_, *u*), where *s*_*t*_ and *h*_*t*−1_ represent the observation at time step *t* and the hidden state at time step *t* − 1, which has information of previous states and acts as a memory. The proposed method is also to be combined with the dueling deep Q-networks (Wang et al., 2016). The dueling deep Q-networks is a neural network architecture designed for value-based RL that has two streams in the computation of the state-action value. One stream is for approximating the value function *V*(*s*) and the other is for approximating the advantage function *A*(*s*, *u*). The value function *V*(*s*) depends only on state and presents how good a state is. The advantage function *A*(*s*, *u*) depends on both state and action and presents how advantageous it is to take an action *u* in comparison to the other actions at the given state *s*. The value and the advantage are merged to get the final state-action value *Q*(*s*, *u*) as follows

(1) \begin{eqnarray*}Q(s,u)=V(s)+A(s,u)- \frac{\sum _{{u}^{{^{\prime}}}}A(s,{u}^{{^{\prime}}})}{N} ,\end{eqnarray*}

where *u*′ represents each possible action and N is the number of actions. In this paper, the dueling deep Q-networks is combined with the RNN to handle the action-observation history used as the input of the policy. In the architecture of dueling deep Q-networks with the RNN, *e.g.*, Dueling DRQN, the RNN is inserted right before the crossroad of streams of computation. The dueling DRQN is compared with the DRQN as an ablation study in ‘Simulation Results’.

In the following subsections, other methods relevant to comparisons are presented. In this paper, we focus on methods that can be combined with off-policy value-based algorithms and focus on the maximization of a joint state-action value, trying to assign proper credit to individual agents on the shared reward received.

### Counterfactual multi-agent policy gradients

Counterfactual multi-agent (COMA), introduced by Foerster et al. (2018), utilizes a single centralized critic to train decentralized actors and deals with the challenge of the multi-agent credit assignment problem. In the cooperative environments that are the main target for the COMA, it is difficult to determine the contribution of each agent to the shared collective reward received by the team. The centralized critic has access to the global state and the actions of the agent to model the joint state-action value function.

### Value decomposition network

The value decomposition network (VDN) (Sunehag et al., 2017) aims at learning a joint-action value function *Q*_*tot*_(*τ*, **u**), where *τ* is a joint-action observation history and **u** is a joint-action. The *Q*_*tot*_(*τ*, **u**) can be expressed as a sum of *a*-th agent’s individual value functions *Q*_*a*_(*τ*^*a*^, *u*^*a*^; *θ*^*a*^) as follow

(2) \begin{eqnarray*}{Q}_{tot}(\tau ,\mathbf{u})=\sum _{a=1}^{n}{Q}_{a}({\tau }^{a},{u}^{a};{\theta }^{a}),\end{eqnarray*}

where each *Q*_*a*_(*τ*^*a*^, *u*^*a*^; *θ*^*a*^) is a utility function of the *a*-th agent and *θ*^*a*^ is the policy of the *a*-th agent. The loss function for the VDN is the same as that of the deep Q-network (DQN) (Mnih et al., 2015), where *Q* is replaced by *Q*_*tot*_(*τ*, **u**).

### QMIX

QMIX (Rashid et al., 2020) is a deep multi-agent reinforcement learning method to be trained using CTDE. It uses the additional global state information that is the input of a mixing network. The QMIX is trained to minimize the loss, just like the VDN (Sunehag et al., 2017), given as

(3) \begin{eqnarray*}\mathcal{L}(\theta )=\sum _{i=1}^{b}[({y}_{i}^{tot}-{Q}_{tot}(\tau ,\mathbf{u},s;\theta ))^{2}],\end{eqnarray*}

where b is the batch size of transitions sampled from the replay buffer and *Q*_*tot*_ is output of the mixing network and the target ${y}_{i}^{tot}=r+\gamma ma{x}_{{\mathbf{u}}^{{^{\prime}}}}{Q}_{tot}({\tau }^{{^{\prime}}},{\mathbf{u}}^{{^{\prime}}},{s}^{{^{\prime}}};{\theta }^{-})$, and *θ*^−^ are the parameters of a target network. The QMIX allows learning of joint-action-value functions, which are equivalent to the composition of optimal Q-values of each agent. This is achieved by imposing a monotonicity constraint on the mixing network. Monotonicity can be enforced by the constraint on the relationship between *Q*_*tot*_ and individual *Q* value functions, given as

(4) \begin{eqnarray*}{Q}_{a}: \frac{\partial {Q}_{tot}}{\partial {Q}_{a}} \geq 0,\forall a\in A.\end{eqnarray*}

## Proposed Method

In heterogeneous multi-agent reinforcement learning, the main challenge can be described as how to provide an optimal policy and maximize cooperative behavior in a heterogeneous multi-agent environment. In this scenario, the agents act independently and maximize not only the individual reward but also a shared reward. To tackle this problem, a novel training method called two-stage heterogeneous centralized training is proposed and described in this section and to be applied to 5 *versus* 5 AI robot soccer.

### MARL structure for AI robot soccer

**Figure 1:**
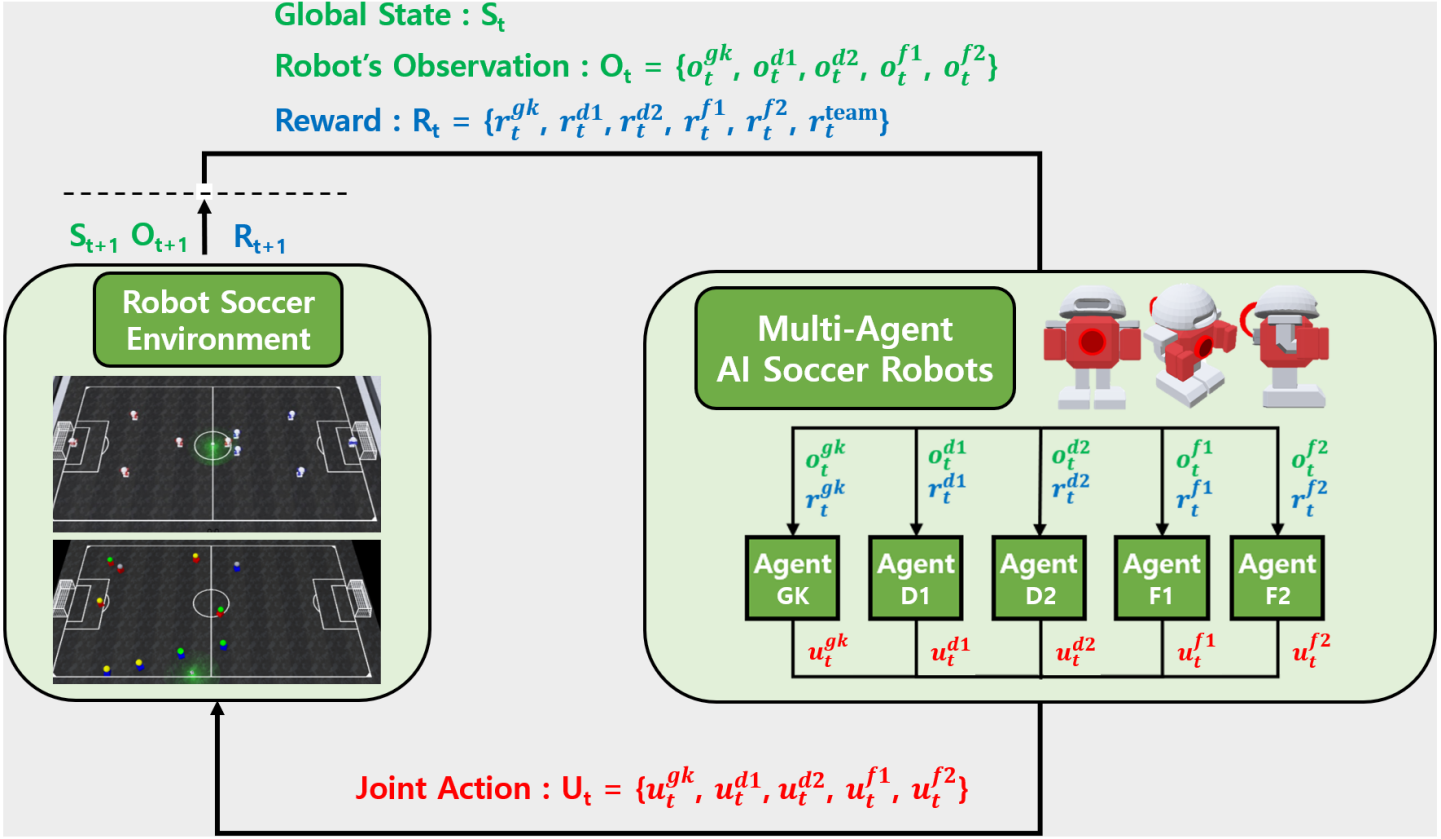
MARL structure for AI robot soccer.

The MARL structure in 5 *versus* 5 AI robot soccer is presented in Fig. 1. In the AI robot soccer each robot has its role. The roles are goalkeeper, defender 1, defender 2, forward 1, and forward 2 which are denoted as GK(gk), D1(d1), D2(d2), F1(f1), and F2(f2), respectively. Each robot has individual observations and individual rewards according to its role in soccer game. Each robot receives its individual observation ${o}_{t}^{role},role\in \{ gk,d1,d2,f1,f2\} $ at each time step *t* and selects its action ${u}_{t}^{role}$ according to a policy network which is trying to maximizing individual role rewards ${r}_{t}^{role}$ and team reward ${r}_{t}^{team}$. The policy network also takes into consideration past individual observations and actions taken. The concatenation of individual actions of the 5 robots forms a joint-action set *U*_*t*_. By performing this joint-action in the AI robot soccer environment, the simulator calculates the next global state *S*_*t*+1_, robot observation *O*_*t*+1_, and reward *R*_*t*+1_. It is noted that the global state is available only during training.

### TSHCT architecture

**Figure 2:**
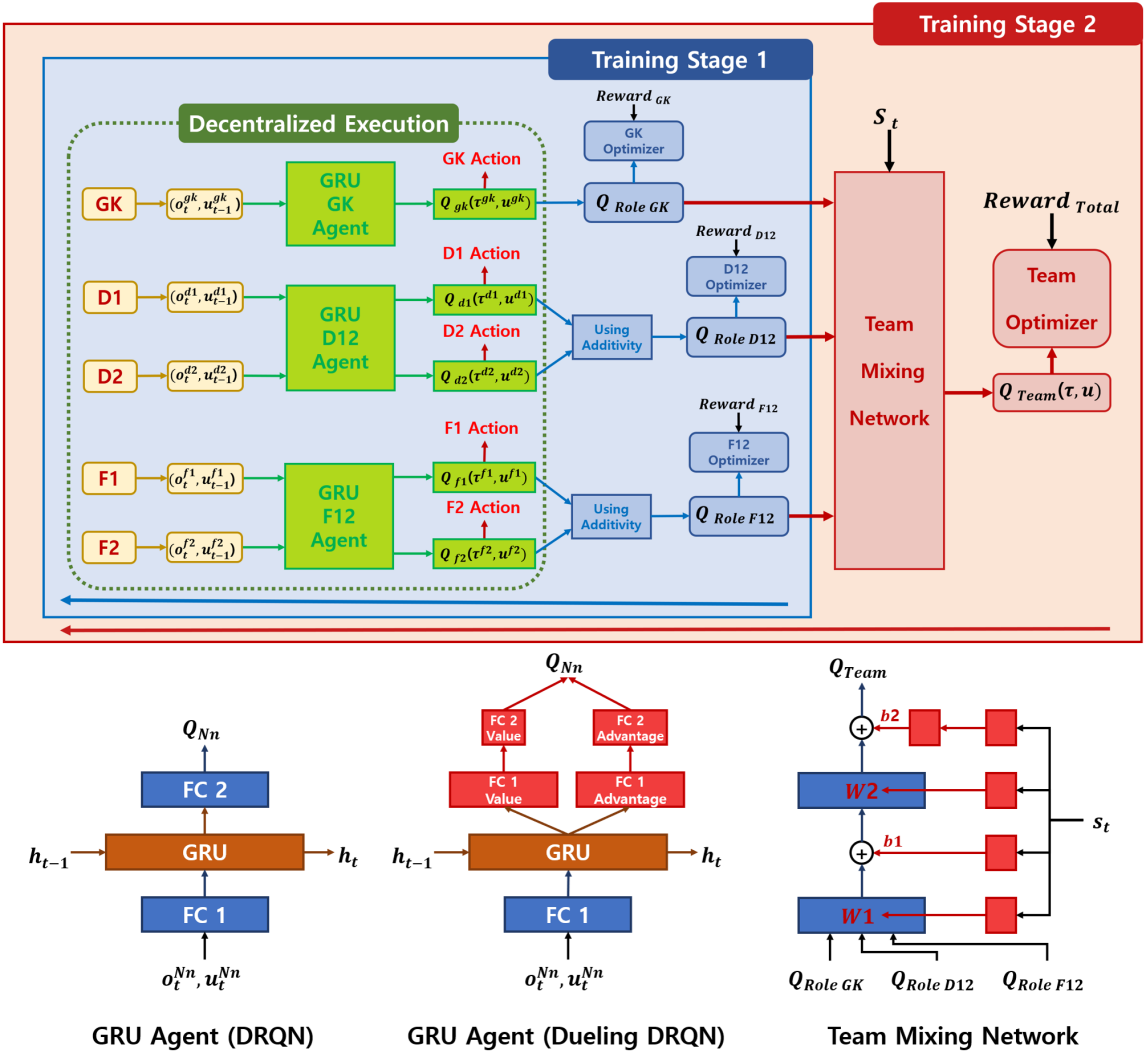
Overall architecture of two-stage heterogeneous centralized training. In the first stage, the agents are trained using their individual role rewards, goalkeeper reward, defender reward, and forward reward. A shared policy is used by defenders and by forwards. In the second stage, the agents are trained using a collective team reward. The global state of the environment *s*_*t*_ is also used as an input of the team mixing network, following the structure of a hypernetwork (Ha, Dai & Le, 2016).

As shown in Fig. 2, the training procedure is divided into two stages. In the first stage, agents of the same type (homogeneous agents, *e.g.*, two agents as defenders) are trained. Decentralized execution is used during inference and a shared policy is used by the agents of the same type. In the second training stage, all heterogeneous agents are trained jointly. These two stages are executed in a serial learning structure.

To model each agent’s policy, the structure of DQN with gated recurrent unit(GRU) (Chung et al., 2014) or the structure of Dueling Q-Networks with GRU is used in the experiments. The policy network receives as input 40 subsequential frames with the current individual observation of the agent ${o}_{t}^{({N}_{n})}$ and the last action chosen ${u}_{(t-1)}^{({N}_{n})}$, where *N*_*n*_ is the *n*-th agent of the *N*-role (type). The output of the policy network is the state-action value *Q*_*N*_*n*__. The action with the highest Q-value is chosen at each time step with epsilon greedy exploration.

In training stage 1, the *Q*_(*RoleN*)_∀*N* ∈ {*GK*(*goalkeeper*), *D*12(*defenders*), *F*12(*forwards*)} is calculated by adding Q-values *Q*_*N*_*n*__ from the homogeneous agent network. In training stage 2, the team mixing network combines the individual role rewards into the shared collective reward. The mixing network is modeled as a hypernetwork (Ha, Dai & Le, 2016), using feed-forward layers. The hypernetwork is conditioned on the global state *S*_*t*_ of the environment and takes the values of *Q*_(*RoleGK*)_, *Q*_(*RoleD*12)_, and *Q*_(*RoleF*12)_ produced in training stage 1 as inputs. The output of the mixing network is *Q*_*Team*_.

### TSHCT learning equations

The proposed method is used to minimize the losses through the entire training. In training stage 1, each role optimizer updates the weights of the policy network to minimize the loss ${\mathcal{L}}_{RoleN}(\mathrm{&theta;})$ in relation to the target *y*^*RoleN*^. The target *y*^*RoleN*^ is calculated based on the Bellman equation (Bellman, 1954) with the sum of the individual role rewards *Reward*_*RoleN*_ for the current time step and the Q-value estimated for the next state. The target and the loss are given as follows

(5) \begin{eqnarray*}& & {y}^{RoleN}=Rewar{d}_{RoleN}+\gamma ma{x}_{{u}^{{^{\prime}}}}{Q}_{RoleN}({\tau }^{{^{\prime}}},{u}^{{^{\prime}}},{s}^{{^{\prime}}};{\theta }^{-}),\nonumber\\\displaystyle & & {\mathcal{L}}_{RoleN}(\theta )=\sum _{i=1}^{b}[({y}_{i}^{RoleN}-{Q}_{RoleN}(\tau ,u,s;\theta ))^{2}],\end{eqnarray*}

where *γ* and *θ*^−^ are the parameters of a target network, the discount factor and policy, similar to the ones presented in DQN (Mnih et al., 2015) to stabilize the training procedure and *b* is the batch size of episodes sampled from the replay buffer. In training stage 2, the team optimizer updates the weights of mixing network and policy networks to minimize the team loss in relation to the team target *y*^*Team*^ calculated with the total shared reward *Reward*_*Total*_, which is the sum of sparse cooperative team rewards and dense individual role rewards. The team loss ${\mathcal{L}}_{Team}(\mathrm{&theta;})$ is given as follows

(6) \begin{eqnarray*}& & {y}^{Team}=Rewar{d}_{Total}+\gamma ma{x}_{{\mathbf{u}}^{{^{\prime}}}}{Q}_{Team}({\tau }^{{^{\prime}}},{\mathbf{u}}^{{^{\prime}}},{s}^{{^{\prime}}};{\theta }^{-}),\nonumber\\\displaystyle & & {\mathcal{L}}_{Team}(\theta )=\sum _{i=1}^{b}[({y}_{i}^{Team}-{Q}_{Team}(\tau ,\mathbf{u},s;\theta ))^{2}].\end{eqnarray*}

Equations (5) and (6) are analogous to the minimum squared loss used in Mnih et al. (2015). Using additivity (Sunehag et al., 2017) and monotonicity (Rashid et al., 2020), the TSHCT trains heterogeneous agents by maximizing *Q*_*Team*_ in stage 2, while learning multiple roles by maximizing the Q-value of each individual role *Q*_(*RoleGK*)_, *Q*_(*RoleD*12)_, and *Q*_(*RoleF*12)_ in stage 1.

### TSHCT curriculum learning through self-play

To train a robust policy in a competitive-cooperative scenario that can work well against multi-agent in the opponent team, curriculum learning is needed. In this paper, we use self-play as a form of the implicit curriculum with the objective of learning robust AI robot soccer strategies. The implicit self-play curriculum is implemented by updating the opponent team when the number of episodes reaches a particular number. The opponent team is kept updated and reference policies take turns. Using self-play, it is possible to keep the opponent team at an appropriate level of competitivity, not too strong so that the policy allows good behavior and not too easy so that the policy avoids weak behaviors. The soccer strategy learned through self-play tends to lead to acceptable game performance (Heinrich, Lanctot & Silver, 2015; Lanctot et al., 2017; Silver et al., 2017) as the result of the automated curriculum.

## Simulation Results

In this section, the MARL environment used in the experiments and the results obtained by the TSHCT and other baseline methods are described.

### AI robot soccer MARL environment

**Figure 3:**
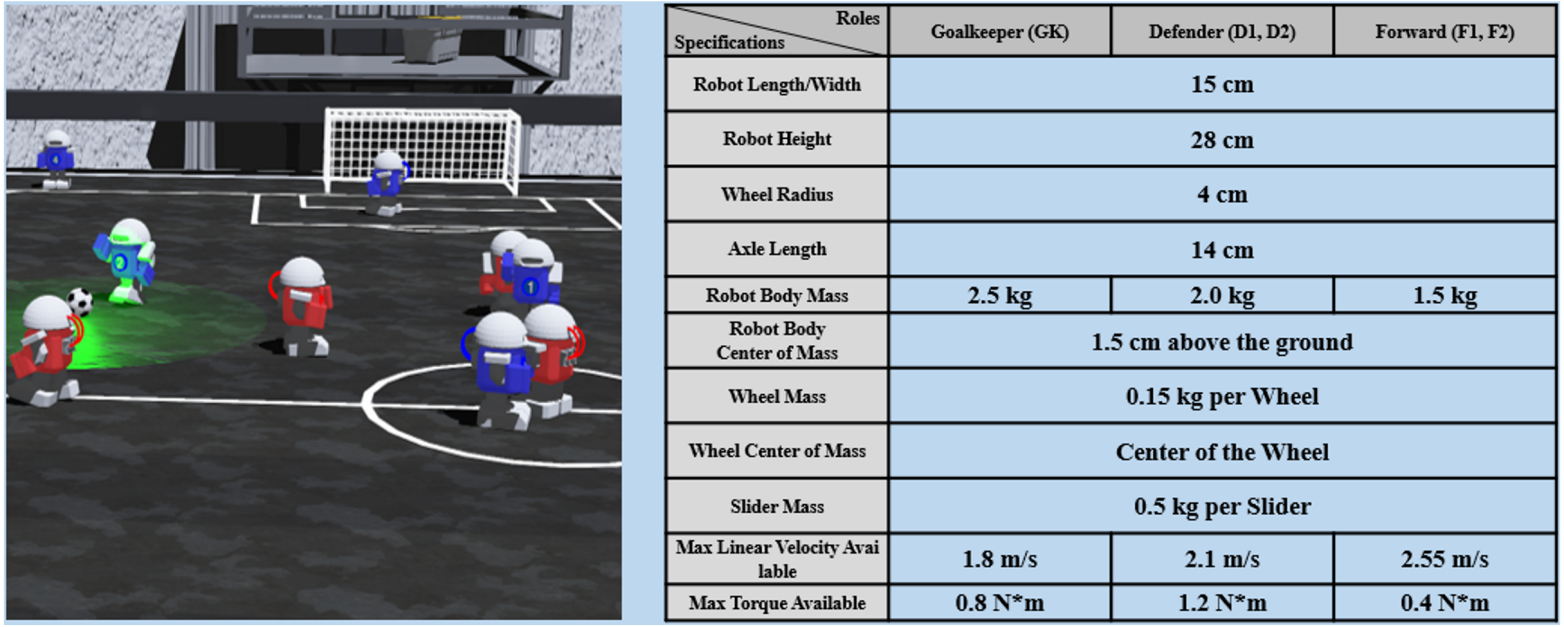
Specifications of the AI robot soccer environment. Robots with different roles, goalkeeper, defender, or forward, have different mass, maximum linear velocity, and maximum torque.

To demonstrate the performance of the TSHCT, experiments are conducted in an AI robot soccer environment with specifications presented in Fig. 3, which is developed with Webots robot simulation software (Michel, 2004) and based on the environment described in Hong et al. (2021). In this AI Soccer simulation game, two teams compete similarly to a real soccer game, trying to kick the ball into the opponent’s goal area to score and to win the game against the opponent team. In each team, there are 5 robots with 3 different roles (one goalkeeper, two defenders, and two forwards). The AI robot soccer game is divided into two 5 minute-long halves. For training, the game is divided up into episodes of 40 sequential frames. An episode is over whenever 40 sequential frames are processed.

#### Global state and observations

The global state, available only during centralized training and used as input to the mixing network, contains information of all the soccer robots and the ball. Specifically, the state vector contains the coordinates and orientations of all soccer robots, including robots of the opponent team, and the ball coordinates. The coordinates are relative to the center of the field (origin). The individual local observations of each robot are their relative positions in the field and relative distances and orientations to other robots and to the ball within their range of view. These observations are used as inputs of the policy networks.

#### Action

The basic actions committed by the robots are move, jump and kick. They are achieved by giving continuous control variables to the feet and legs. To achieve these behaviors a discrete set of 20 actions is designed which is allowed to be taken by the agent at each time step. A discrete set of actions is used so that the DRQN and the Dueling DRQN can be used as the off-policy value-based algorithms for the experiments. The discrete action set consists of actions of forward motion, backward motion, 6 directions of forward turns, 4 directions of backward turns, clockwise and counterclockwise turns, 2 kinds of forward turn combined with kick, 2 kinds of forward motion combined with kick, stop combined with kick, and stop.

#### Reward

To train AI soccer robots to perform their roles and cooperative behavior, individual role rewards and a shared team reward are defined. Individual role rewards are a combination of dense rewards associated with two pieces of information. One is the ball information relative to the robot, such as distance, velocity, and angle. The other is the information of the expected position which is defined for each role, *i.e.,* default position where the robot should be to play its role. The team reward is a combination of a sparse reward related to scoring and dense rewards related to the distance and velocity between the ball and the opponent’s goal.

Equations (7) and (8) show the mathematical modeling of the individual role reward and the team reward. In Eq. (7), *d*_*rp*_ is the distance between the robot and its expected role position, *θ*_*rb*_ and *v*_*rb*_ are the relative angle and relative velocity between the robot and the ball, *d*_*bg*,*pre*/*cur*_ is the distance between the ball and the opponent goal center at previous/current time step, and *isTouch* is a boolean that is true when the robot touched the ball within the last 10 time steps. In Eq. (8), *d*_*bg*_ and *v*_*bg*_ are distance and velocity between the ball and the opponent goal center and *isScore* is 100 if the team scored against the opponent team.

(7) \begin{eqnarray*}{r}^{role}={e}^{-{d}_{rp}}+0.5{e}^{-{\theta }_{rb}}+0.5(1-{e}^{-{v}_{rb}})+50({d}_{bg,pre}-{d}_{bg,cur})\times isTouch.\end{eqnarray*}

(8) \begin{eqnarray*}{r}^{team}=5{e}^{-{d}_{bg}}+5(1-{e}^{-{v}_{bg}})+isScore.\end{eqnarray*}

### Network hyperparameters

The neural network hyperparameters used in the experiments are as follows

• DRQN architecture: 2 layers with 128 hidden units, 1 layer of GRU with 128 hidden units, and ReLU non-linearities.
• Dueling DRQN architecture: 1 layer with 128 hidden units, 1 layer of GRU with 128 hidden units, 2 layer with 128 hidden units for value prediction, 2 layer with 128 hidden units for advantage prediction, and ReLU non-linearities.
• Mixing network architecture: 1 layer of mixing network with 32 hidden units, 2 layers of hypernetworks with 32 hidden units, and ReLU non-linearities.
• ADAM optimizer (Kingma & Ba, 2014) with learning rate set to 4 × 10^−5^ for both policy and mixing networks.
• Discount factor *γ* set to 0.99.
• Target networks updated every 16,000 iterations.
• Epsilon used for exploration decreased by 0.025 every 10^4^ iterations until it is kept at 0.05 at the end of training.
• Buffer size set to store 5 × 10^3^ episodes.
• Batch size set to 64.

###  Results

#### Evaluation of TSHCT and baselines, COMA, VDN, and QMIX

In this section, the evaluation of the TSHCT and baseline methods, COMA, VDN, and QMIX, are presented. The proposed method and baseline methods are trained for a total of 200k episodes using epsilon greedy exploration with self-play. The evaluation is conducted by comparing the performances of 4 algorithms, TSHCT, COMA, VDN, and QMIX. The performances are measured by matches against three evaluation teams, noted as Evaluation Team 1, 2, and 3. As the result of the evaluation, comparisons of rewards and score-concede rates are given. The “score” term means a goal scored by own team while the “concede” term representss a goal scored by the opponent team. The score-concede rate is defined as the percentage of the number of scores divided by the sum of the number of scoring and conceding.

**Figure 4:**
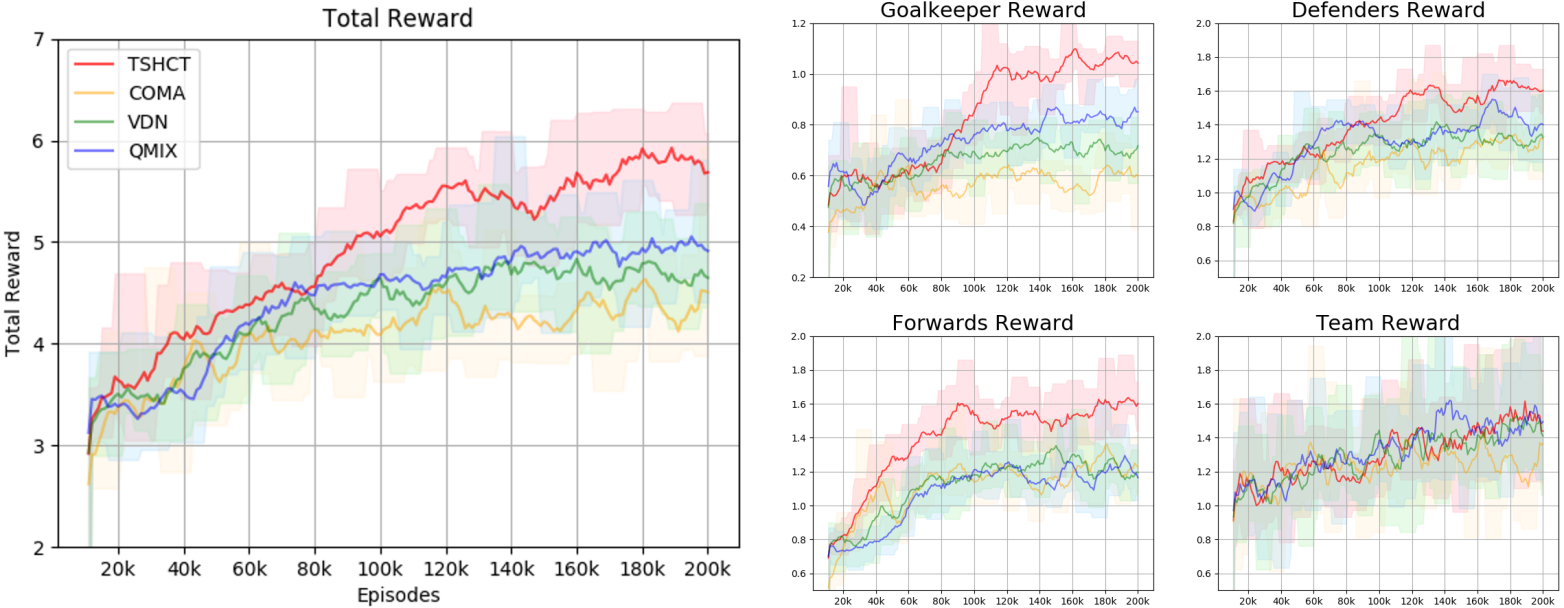
Total reward obtained during training by TSHCT, COMA, VDN, and QMIX. It is evaluated against Evaluation Team 1.

**Figure 5:**
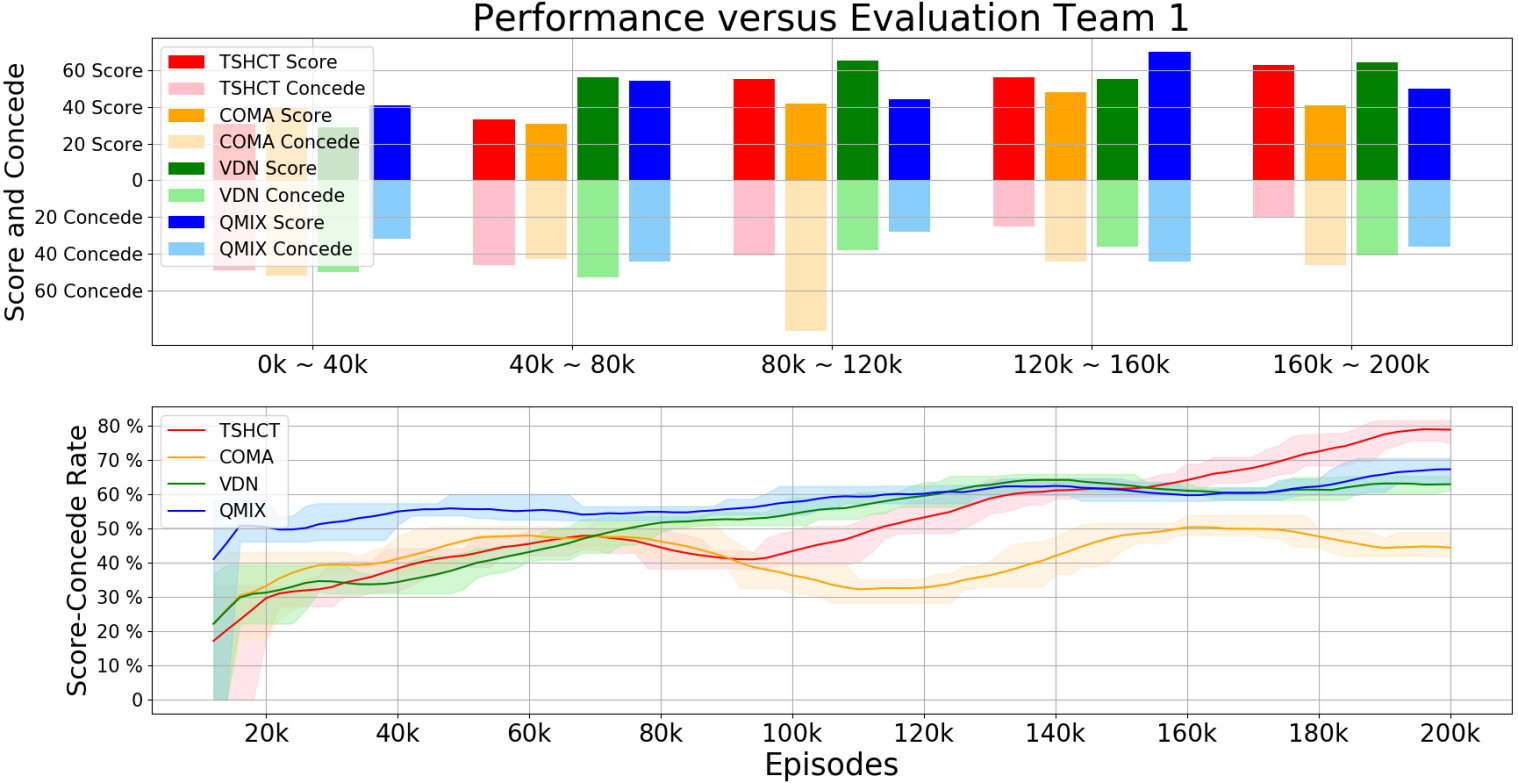
Comparison of score, concede, and score-concede rate obtained during training by TSHCT, COMA, VDN, and QMIX. It is evaluated against Evaluation Team 1.

In the first evaluation, the performances of the TSHCT and the baselines are obtained by playing against the Evaluation Team 1, which is a team trained for 200k episodes with COMA. The experimental result shows that the TSHCT is superior to COMA, VDN, and QMIX algorithms after 80k episodes, as shown in Fig. 4, where the total reward is defined as the sum of three individual rewards and the team reward. When the maximum average total reward is defined as the maximum value of the average of total reward of sequential 10,000 episodes, the maximum average total rewards of TSHCT, COMA, VDN, and QMIX are 5.92, 4.63, 4.83, and 5.05, respectively. The score-concede rate is defined as the maximum value of the averages of score-concede rates obtained over 10 sequential games. The score-concede rates of TSHCT, COMA, VDN, and QMIX are 79.01%, 50.40%, 64.21%, and 67.30%, respectively, as shown in Fig. 5. It is observed that the TSHCT improves the score-concede rate by 28.61% as compared to that of COMA.

**Figure 6:**
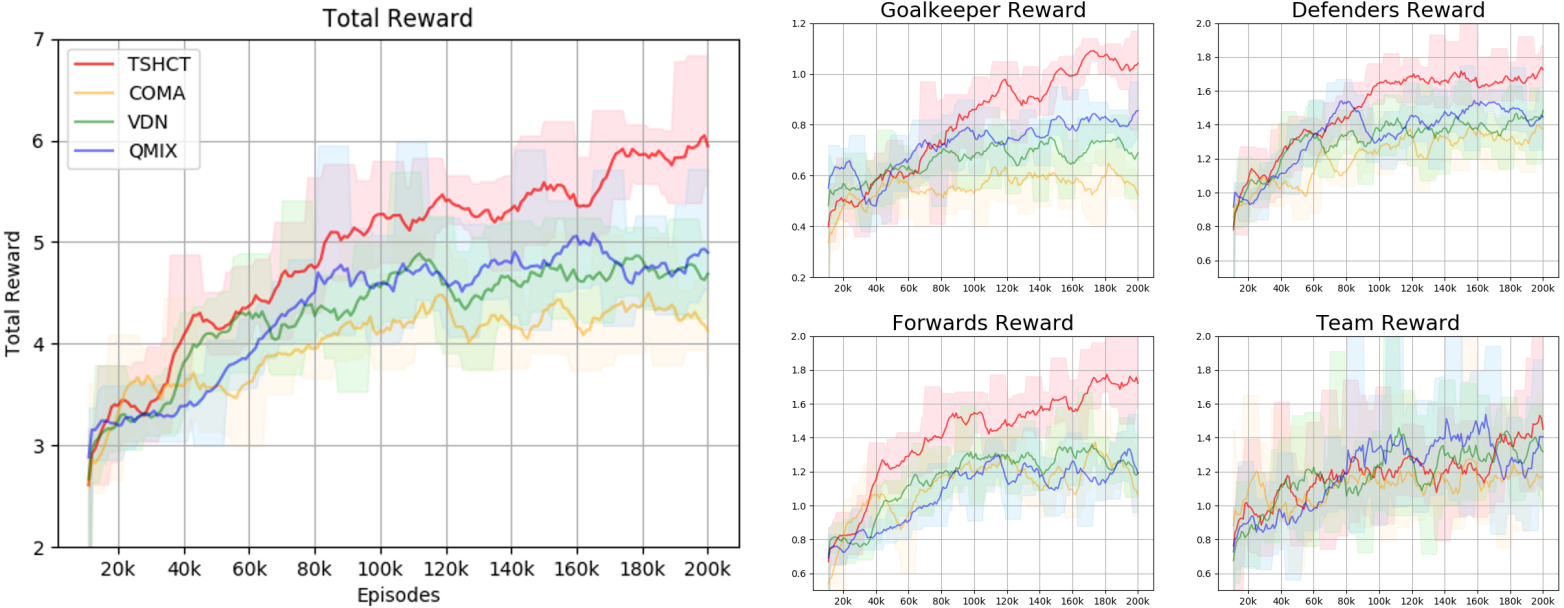
Total reward obtained during training by TSHCT, COMA, VDN, and QMIX. It is evaluated against Evaluation Team 2.

**Figure 7:**
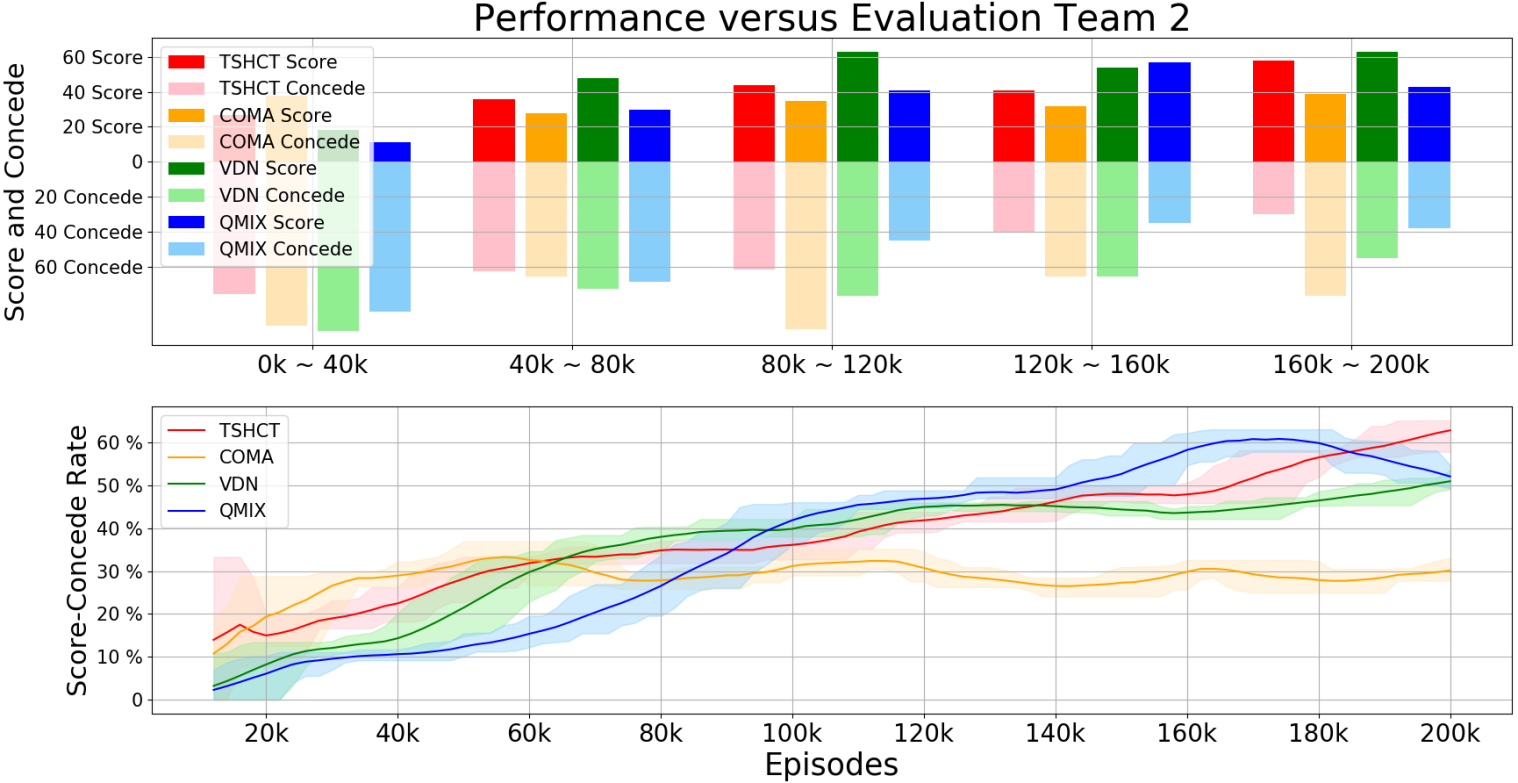
Comparisons of score, concede, and score-concede rate obtained during training by TSHCT, COMA, VDN, and QMIX. The score, concede, and score-concede rate are evaluated against Evaluation Team 2.

For the second evaluation, the performances of the TSHCT and the baselines are measured by playing against the Evaluation Team 2, which is a team trained for 200k episodes with VDN. Experiment results presented in Fig. 6 show that the TSHCT is superior to the baseline algorithms after 80k episodes. The maximum average total rewards of TSHCT, COMA, VDN, and QMIX are 6.05, 4.50, 4.89, and 5.08, respectively. The maximum averages of score-concede rate of TSHCT, COMA, VDN, and QMIX are 62.85%, 32.27%, 50.97%, and 60.85%, respectively, as shown in Fig. 7. It is observed that the TSHCT improved the score-concede rate by 11.88% as compared to that of VDN.

**Figure 8:**
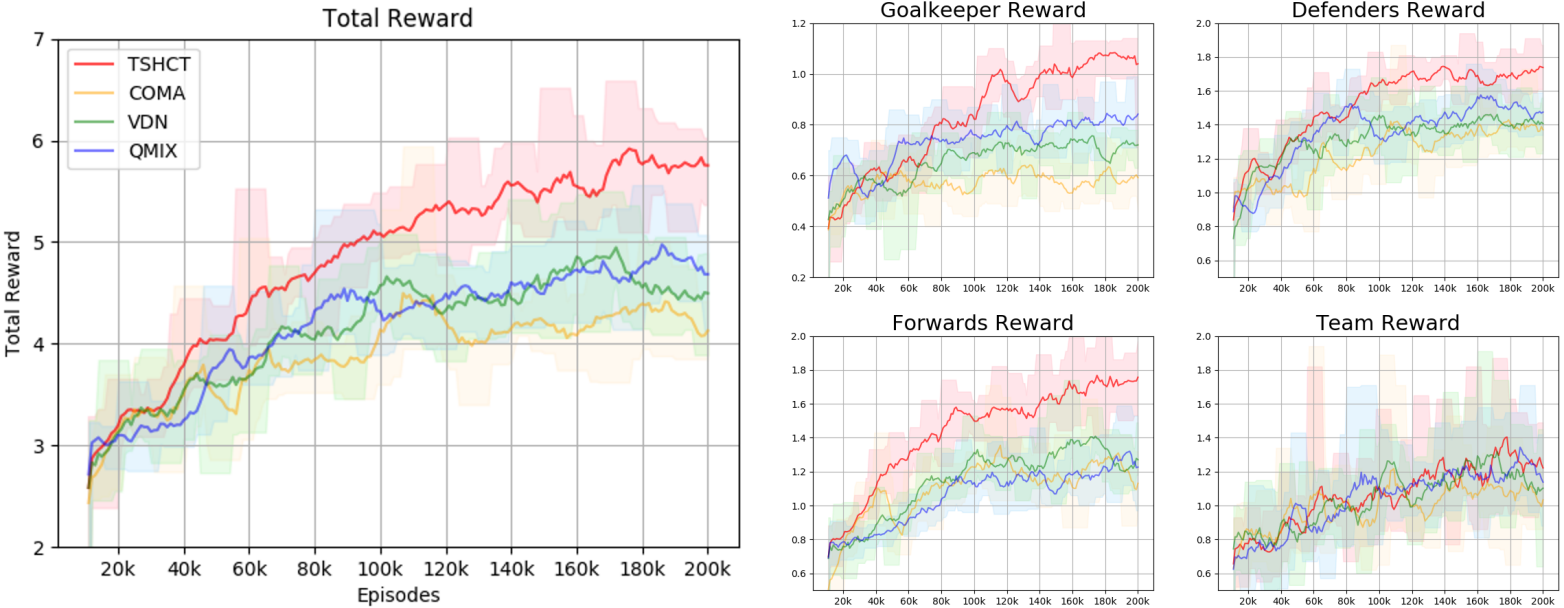
Total reward obtained during training by TSHCT, COMA, VDN, and QMIX evaluated against Evaluation Team 3.

**Figure 9:**
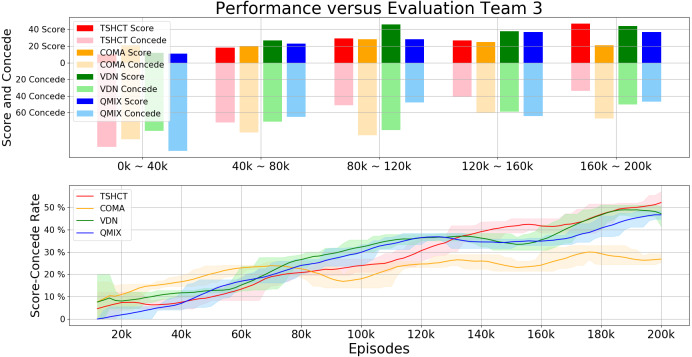
Comparison of score, concede, and score-concede rate obtained during training by TSHCT, COMA, VDN, and QMIX. The score, concede, and score-concede rate are evaluated against Evaluation Team 3.

For the third evaluation, the performances of the TSHCT and the baselines are obtained by playing against the Evaluation Team 3, which is a team trained for 200k episodes with QMIX. Experiment results show that TSHCT outperforms the baseline algorithms after 60k episodes, as shown in Fig. 8. The maximum average total rewards of TSHCT, COMA, VDN, and QMIX are 5.92, 4.50, 4.95, and 4.98, respectively. The maximum averages of score-concede rate of TSHCT, COMA, VDN, and QMIX are 52.08%, 29.99%, 48.84%, and 46.63%, respectively, as shown in Fig. 9. It is seen that the TSHCT improved the performance by 5.45% as compared to that of QMIX. It is important to mention that QMIX is the algorithm with the best performance when compared with the other baseline methods, VDN and COMA. s

The final performances of the policies trained by the proposed method and the baseline methods are compared by conducting 10 min matches. Table 1 summarizes the results and statistics of these matches.

**Table 1:** Results and statistics of evaluation matches for TSHCT against the baseline methods.

**TSHCT**		*vs* **COMA**	*vs* **VDN**	*vs* **QMIX**
100K episodes trained policy	Score	7.09 ± 1.83	3.92 ± 1.38	3.82 ± 1.70
	Concede	3.27 ± 1.54	4.23 ± 2.04	4.55 ± 0.89
	Score difference	3.82	−0.30	−0.73
	Score concede rate	68.4%	48.1%	45.6%
	Winning rate	100%	50%	20%
200k episodes trained Policy	Score	5.55 ± 2.23	5.00 ± 1.13	3.45 ± 1.44
	Concede	2.18 ± 1.59	2.82 ± 1.70	3.00 ± 1.41
	Score difference	3.37	2.18	0.45
	Score concede rate	71.8%	63.9%	53.5%
	Winning rate	100%	90%	80%

#### Ablation study: DRQN *vs* dueling DRQN

In AI robot soccer, several different sequences of actions can lead to similar reward values. From this observation, an ablation study is conducted by combining the TSHCT with dueling Q-network. Dueling Q-network often leads to better policy in the presence of distinct actions leading to similar reward values (Wang et al., 2016). In this ablation study, the traditional dueling Q-network architecture is combined with the RNN, which is named here as Dueling DRQN. The proposed method combined with the Dueling DRQN is compared with the TSHCT combined with the DRQN. The TSHCT with Dueling DRQN is trained with 200k episodes using epsilon greedy exploration with self-play, similar to the cases shown in Figs. 5, 7 and 9. For comparisons of rewards and score-concede rates, game matches between the team trained by the TSHCT with DRQN, TSHCT-DRQN, and the team trained by the TSHCT with Dueling DRQN, TSHCT-Dueling DRQN, are conducted. The results of these matches are presented in Table 2.

**Table 2:** Results and statistics of evaluation matches for TSHCT-Dueling DRQN against TSHCT-DRQN.

TSHCT-Dueling DRQN		*vs* TSHCT-DRQN	
		**100k episodes trained policy**	**200k episodes trained policy**
100k episodes trained policy	Score	5.09 ± 2.07	3.91 ± 1.78
	Concede	3.82 ± 2.03	4.64 ± 2.19
	Score difference	1.27	−0.73
	Score concede rate	57.1%	45.7%
	Winning rate	60%	30%
100k episodes trained policy	Score	8.36 ± 2.64	5.82 ± 2.48
	Concede	1.55 ± 1.30	2.27 ± 1.14
	Score difference	6.81	3.55
	Score concede Rate	84.4%	71.9%
	Winning rate	100%	80%

**Figure 10:**
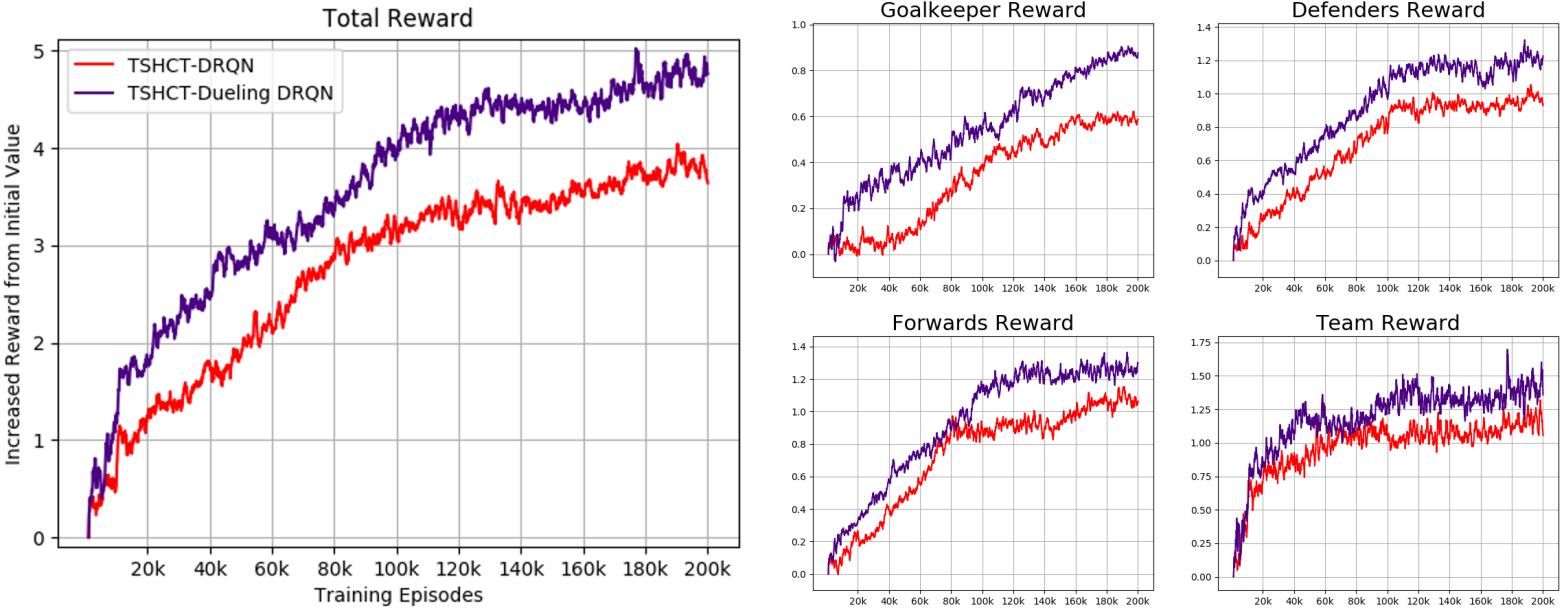
Rewards of TSHCT with DRQN and Dueling DRQN during training for 200k episodes with self-play.

**Figure 11:**
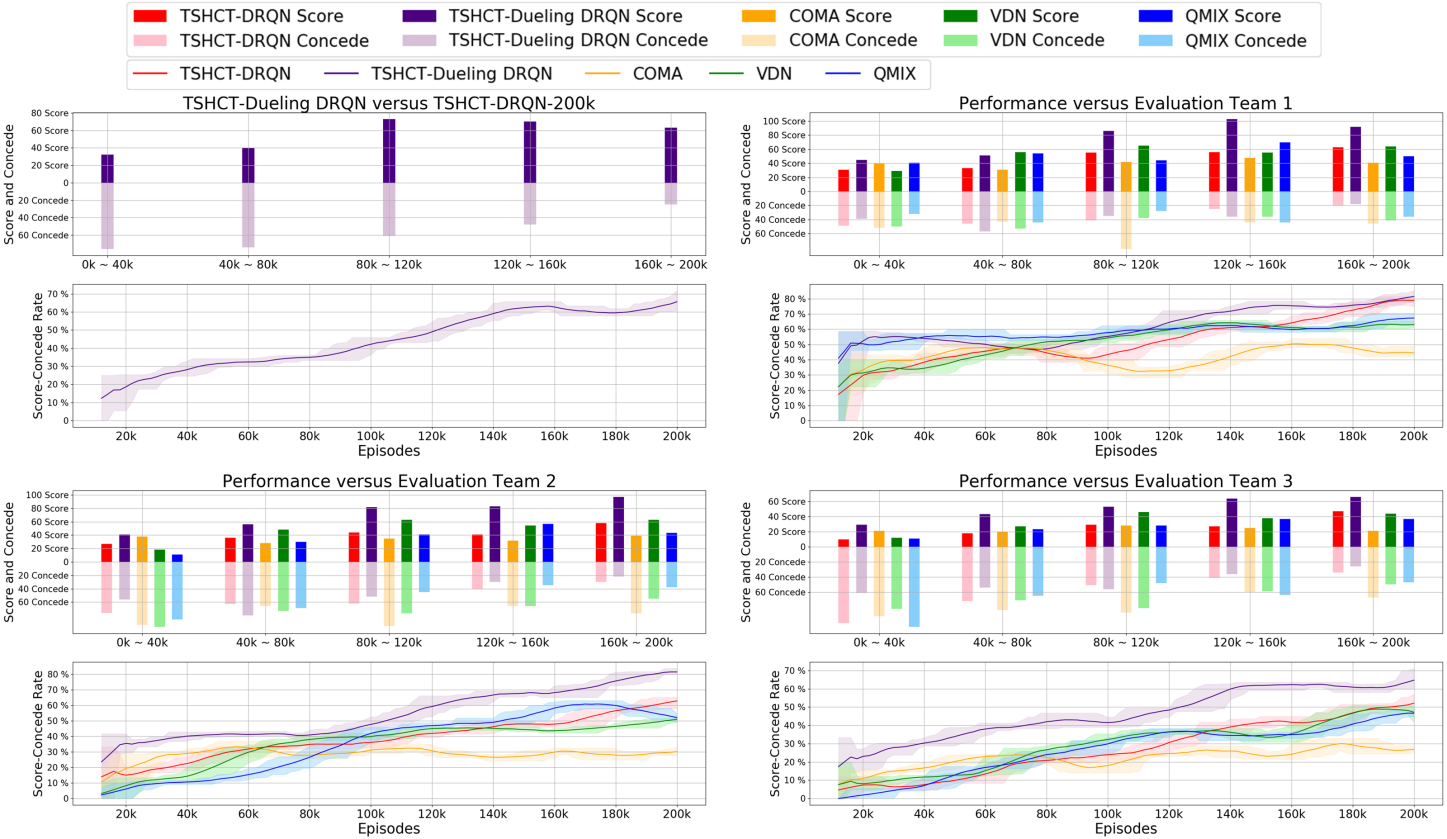
Comparison of score, concede, and score-concede rate obtained during training by TSHCT-DRQN, TSHCT-Dueling DRQN, COMA, VDN, and QMIX. The score, concede, and score-concede rate are evaluated against a team trained by the TSHCT with DRQN and three evaluation teams.

In Fig. 10, the rewards obtained by the TSHCT with DRQN and the TSHCT with Dueling DRQN are presented. Figure 10 shows the increasing trends of rewards. It is seen that the TSHCT with Dueling DRQN leads to a higher total reward as compared to the TSHCT with DRQN. The maximum average score-concede rates of the team trained by the TSHCT with Dueling DRQN against a team trained by the TSHCT with DRQN and three evaluation teams are 65.59%, 81.49%, 81.52%, and 64.67%, respectively, as shown in Fig. 11. The TSHCT with Dueling DRQN demonstrates improved score-concede rates over Evaluation Team 1, 2, and 3 by 2.48%, 18.67%, and 12.59% as compared to that obtained by the TSHCT with DRQN.

The policies trained by the TSHCT combined with DRQN and by the TSHCT combined with Dueling DRQN are compared with game results. Table 2 lists the results of these evaluation matches. For policies trained with the same number of training episodes, the TSHCT combined with Dueling DRQN outperforms the TSHCT combined with DRQN, achieving 60% and 80% winning rates with 100k episodes and 200k episodes, respectively. For the cases in which one algorithm is trained with two times the number of episodes of the opponent, *i.e.,* 200k *versus* 100k, the algorithm that was trained for a longer time achieves a higher winning rate. However, even for this case, the trained policy using the TSHCT combined with Dueling DRQN is more robust, achieving a 30% winning rate and a score-concede rate of 45.7%.

### Discussion

Efficient exploration and reward modeling remain a big challenge in complex multi-agent environments. In a game such as robot soccer, using only team rewards, *e.g.*, a sparse score/concede reward or a sparse win/lose reward after the game is finished, is not enough for the agents to learn robust behavior. To deal with this problem, Vinyals et al. (2019) use data from professional players at the beginning of the training in a supervised fashion to train a Starcraft 2 agent. Without this supervised data, it is difficult for the models to achieve a level capable of playing against good players and exploiting game strategies. In this aspect, by the results obtained in the results section, additional information in the form of individual role rewards that can be provided or learned unsupervised improves the policies.

In relation to the improvement of team rewards during training, the results obtained in the simulations indicate that it is difficult to train for cooperative behavior while performing multiple roles. The results obtained by the proposed method and the baseline methods, COMA, VDN, and QMIX, suggest that techniques that assign the contribution of each robot in the reward received as well as the techniques that train individualized roles that lead to stronger agents is needed. This can be addressed by the proposed method using two training stages. The stage 1 induces the learning of individual roles while stage 2 causes the learning of cooperative behavior and maximizing team rewards. From the observation of graphs of the individual role rewards, as shown by reward plots in Figs. 4, 6 and 8, the TSHCT achieves role rewards higher than those obtained by other algorithms. As robot soccer is a game played against an opponent team, the main objective, more than having high rewards during training, is to train multi-agent that performs well against opponents. Observing the matches against evaluation teams, as shown in Figs. 5, 7 and Fig. 9, it is noted that the proposed method is able to achieve substantially higher score-concede rate when compared with other methods. These results suggest that the proposed method in general works better than other methods. In the aspect of computational load, among the proposed method and the baselines, the proposed method takes the second-longest time to train the team for the same number of iterations because of its two stages.

In AI robot soccer, the policies are trained to maximize both individual role rewards and a shared team reward. Individual role rewards are designed for the robots to learn their roles, specifically to learn how to position and to learn how to control the ball to perform passing and shooting. Team rewards are designed for the team to learn how to score against the opponent team, avoid conceding, and also learn how to put pressure on opponent robots during the game (keeping the ball near the opponent goal area as much as possible during the game). The results obtained from simulation, using these rewards, have shown that the robots are able to learn individual role rewards while trying to act collaboratively. The GK learns to move to protect the goal against kicks of the opponent team while trying to kick away if the ball is reachable. The defenders act mostly if the ball is in the own field and try to recover the ball and kick the ball away from goal. When the ball is in the opponent field, defenders mostly try to position themselves in the field to avoid counter-attacks. Forwards are the most active players in the trained policies, trying to always be near the ball and kick the ball along right direction into the opponent’s goal.

It is important to mention that, despite the results being obtained only in a simulated environment, the final goal of the RL approaches is to transfer the policy in a simulation to a real world scenario, such as playing a real robot competition in the RoboCup (Kitano et al., 1995) contest. It is necessary to create a framework with the sensors available in the real robots in real-time so that the work learned by simulation can be transferred to real robots without the need of re-training or with little re-training. Important research works have already been investigated while transferring the results obtained from simulation to real robots (Peng et al., 2018). To train robust models, the most important aspects are to respect the partially observable modeling of the robot soccer environment and to consider the variability of real world scenarios. For such purpose, noise that affects the state, the action, and the physics modeling should be added to the simulation environment so that less fine tuning is needed when deploying the trained policy.

## Conclusion

This paper deals with multi-agent reinforcement learning with heterogeneous agents. The classic way to solve this problem is using the CTDE framework. However, the CTDE framework is less efficient for heterogeneous agents in learning individual behaviors. This paper presents the TSHCT, a novel heterogeneous multi-agent reinforcement learning method that allows heterogeneous agents to learn multiple roles for cooperative behavior. In the proposed method, there are two training stages that are conducted in a serial manner. The first stage is for training individual behavior through maximizing individual role rewards, while the second stage is for training cooperative behavior while maximizing a shared team reward. The experiments are conducted with 5 *versus* 5 AI robot soccer which is relevant to the cooperative-competitive multi-agent environment. The proposed method is compared with other baseline methods that maximize the shared reward to achieve cooperative behavior. The proposed method and baseline methods, COMA, VDN, and QMIX, are combined with value-based algorithms, such as DQN and dueling Q-networks.

Comparisons of total rewards and score-concede rates are presented in the paper. The results show that the TSHCT training method is superior to other baseline algorithms in role training and learning cooperative behavior. The maximum average score-concede rates of the TSHCT in comparison with the COMA, VDN, and QMIX are 79.01%, 62.85%, and 52.08%, respectively, representing the improvement achieved by the TSHCT in competitive AI robot soccer matches.

Because similar action-observation history leads to similar rewards in AI robot soccer, the training process can be unstable. To address this issue, an ablation study comparing the TSHCT combined with Dueling DRQN and DRQN is conducted. The performances of the TSHCT with DRQN and Dueling DRQN are measured by total rewards, score-concede rates, and match results. As a result, the TSHCT combined with Dueling DRQN achieves better performance when compared to the TSHCT combined with DRQN. The maximum average score-concede rate of the TSHCT with Dueling DRQN in comparison with the COMA, VDN, and QMIX are 81.49%, 81.52%, and 64.67%, respectively. This result represents an improvement of 2.48%, 18.67%, and 12.59% as compared to the case of the TSHCT combined with DRQN.

Simulation results show that the TSHCT is able to train an AI robot soccer team effectively, achieving higher individual role rewards and higher total rewards, as compared to other approaches that can be used for training to get cooperative behavior in a multi-agent environment. As future work, this framework is to be combined with actor-critic policy-based multi-agent algorithms that can be applied in environments with continuous actions.

## Additional Information and Declarations

### Data Availability

The following information was supplied regarding data availability: The code files are available at GitHub: https://github.com/ngng9957/TSHCT_dueling

## References

[ref-1] Andrychowicz M, Wolski F, Ray A, Schneider J, Fong R, Welinder P, McGrew B, Tobin J, Abbeel P, Zaremba W (2017). Hindsight experience replay.

[ref-2] Bellman R (1954). The theory of dynamic programming. Technical report, Randcorp santa monica ca.

[ref-3] Berner C, Brockman G, Chan B, Cheung V, Dębiak P, Dennison C, Farhi D, Fischer Q, Hashme S, Hesse C (2019). Dota 2 with large scale deep reinforcement learning.

[ref-4] Chu T, Wang J, Codecà L, Li Z (2019). Multi-agent deep reinforcement learning for large-scale traffic signal control. IEEE Transactions on Intelligent Transportation Systems.

[ref-5] Chung J, Gulcehre C, Cho K, Bengio Y (2014). Empirical evaluation of gated recurrent neural networks on sequence modeling.

[ref-6] Foerster J, Farquhar G, Afouras T, Nardelli N, Whiteson S (2018). Counterfactual multi-agent policy gradients.

[ref-7] Ha D, Dai A, Le QV (2016). Hypernetworks.

[ref-8] Hausknecht M, Stone P (2015). Deep recurrent q-learning for partially observable mdps.

[ref-9] He X, Dai H, Ning P (2015). Improving learning and adaptation in security games by exploiting information asymmetry.

[ref-10] Heinrich J, Lanctot M, Silver D (2015). Fictitious self-play in extensive-form games.

[ref-11] Hochreiter S, Schmidhuber J (1997). Long short-term memory. Neural Computation.

[ref-12] Hong C, Jeong I, Vecchietti LF, Har D, Kim JH (2021). AI world cup: robot soccer-based competitions. IEEE Transactions on Games.

[ref-13] Hwangbo J, Lee J, Dosovitskiy A, Bellicoso D, Tsounis V, Koltun V, Hutter M (2019). Learning agile and dynamic motor skills for legged robots. Science Robotics.

[ref-14] Kim T, Vecchietti LF, Choi K, Lee S, Har D (2020). Machine learning for advanced wireless sensor networks: a review. IEEE Sensors Journal.

[ref-15] Kingma DP, Ba J (2014). Adam: a method for stochastic optimization.

[ref-16] Kitano H, Asada M, Kuniyoshi Y, Noda I, Osawa E (1995). RoboCup: the robot world cup initiative. https://www.robocup.org/.

[ref-17] Klima R, Tuyls K, Oliehoek F (2016). Markov security games: learning in spatial security problems.

[ref-18] Lanctot M, Zambaldi V, Gruslys A, Lazaridou A, Tuyls K, Pérolat J, Silver D, Graepel T (2017). A unified game-theoretic approach to multiagent reinforcement learning.

[ref-19] Lillicrap TP, Hunt JJ, Pritzel A, Heess N, Erez T, Tassa Y, Silver D, Wierstra D (2015). Continuous control with deep reinforcement learning.

[ref-20] Liu S, Lever G, Merel J, Tunyasuvunakool S, Heess N, Graepel T (2019). Emergent coordination through competition.

[ref-21] Lowe R, Wu Y, Tamar A, Harb J, Abbeel P, Mordatch I (2017). Multi-agent actor-critic for mixed cooperative-competitive environments.

[ref-22] Michel O (2004). Cyberbotics Ltd. Webots: professional mobile robot simulation. International Journal of Advanced Robotic Systems.

[ref-23] Mnih V, Badia AP, Mirza M, Graves A, Lillicrap T, Harley T, Silver D, Kavukcuoglu K (2016). Asynchronous methods for deep reinforcement learning.

[ref-24] Mnih V, Kavukcuoglu K, Silver D, Graves A, Antonoglou I, Wierstra D, Riedmiller M (2013). Playing atari with deep reinforcement learning.

[ref-25] Mnih V, Kavukcuoglu K, Silver D, Rusu AA, Veness J, Bellemare MG, Graves A, Riedmiller M, Fidjeland AK, Ostrovski G (2015). Human-level control through deep reinforcement learning. Nature.

[ref-26] Nguyen TT, Nguyen ND, Nahavandi S (2020). Deep reinforcement learning for multiagent systems: a review of challenges, solutions, and applications. IEEE Transactions on Cybernetics.

[ref-27] Oliehoek FA, Amato C (2016). A concise introduction to decentralized POMDPs.

[ref-28] Peng XB, Andrychowicz M, Zaremba W, Abbeel P (2018). Sim-to-real transfer of robotic control with dynamics randomization.

[ref-29] Rashid T, Samvelyan M, De Witt CS, Farquhar G, Foerster J, Whiteson S (2020). Monotonic value function factorisation for deep multi-agent reinforcement learning. Journal of Machine Learning Research.

[ref-30] Sallab AE, Abdou M, Perot E, Yogamani S (2017). Deep reinforcement learning framework for autonomous driving. Electronic Imaging.

[ref-31] Samvelyan M, Rashid T, De Witt CS, Farquhar G, Nardelli N, Rudner TG, Hung C.-M., Torr PH, Foerster J, Whiteson S (2019). The starcraft multi-agent challenge.

[ref-32] Seo M, Vecchietti LF, Lee S, Har D (2019). Rewards prediction-based credit assignment for reinforcement learning with sparse binary rewards. IEEE Access.

[ref-33] Shalev-Shwartz S, Shammah S, Shashua A (2016). Safe, multi-agent, reinforcement learning for autonomous driving.

[ref-34] Silver D, Huang A, Maddison CJ, Guez A, Sifre L, Van Den Driessche G, Schrittwieser J, Antonoglou I, Panneershelvam V, Lanctot M (2016). Mastering the game of Go with deep neural networks and tree search. Nature.

[ref-35] Silver D, Hubert T, Schrittwieser J, Antonoglou I, Lai M, Guez A, Lanctot M, Sifre L, Kumaran D, Graepel T (2018). A general reinforcement learning algorithm that masters chess, shogi, and Go through self-play. Science.

[ref-36] Silver D, Schrittwieser J, Simonyan K, Antonoglou I, Huang A, Guez A, Hubert T, Baker L, Lai M, Bolton A (2017). Mastering the game of go without human knowledge. Nature.

[ref-37] Sunehag P, Lever G, Gruslys A, Czarnecki WM, Zambaldi V, Jaderberg M, Lanctot M, Sonnerat N, Leibo JZ, Tuyls K (2017). Value-decomposition networks for cooperative multi-agent learning.

[ref-38] Vecchietti LF, Kim T, Choi K, Hong J, Har D (2020). Batch prioritization in multigoal reinforcement learning. IEEE Access.

[ref-39] Vecchietti LF, Seo M, Har D (2020). Sampling rate decay in hindsight experience replay for robot control. IEEE Transactions on Cybernetics.

[ref-40] Vinyals O, Babuschkin I, Czarnecki WM, Mathieu M, Dudzik A, Chung J, Choi DH, Powell R, Ewalds T, Georgiev P (2019). Grandmaster level in StarCraft II using multi-agent reinforcement learning. Nature.

[ref-41] Wang Z, Schaul T, Hessel M, Hasselt H, Lanctot M, Freitas N (2016). Dueling network architectures for deep reinforcement learning.

[ref-42] Zhang H, Feng S, Liu C, Ding Y., Zhu Y, Zhou Z, Zhang W, Yu Y, Jin H, Li Z (2019). Cityflow: a multi-agent reinforcement learning environment for large scale city traffic scenario.

